# Relationship between telomere shortening and early subjective depressive symptoms and cognitive complaints in older adults

**DOI:** 10.18632/aging.204533

**Published:** 2023-02-17

**Authors:** Myung-Hoon Han, Eun-Hye Lee, Hyun-Hee Park, Seong Hye Choi, Seong-Ho Koh

**Affiliations:** 1 Department of Neurosurgery, Hanyang University Guri Hospital, Guri 11923, South Korea; 2 Department of Neurology, Hanyang University Guri Hospital, Guri 11923, South Korea; 3 Department of Translational Medicine, Hanyang University Graduate School of Biomedical Science and Engineering, Seoul 04763, South Korea; 4 Department of Neurology, Inha University College of Medicine, Incheon 22332, South Korea

**Keywords:** telomere length, cognitive complaint, depressive symptom, interleukin-6, aging

## Abstract

Telomere length (TL) has been reported to be associated with depression and cognitive impairment in elderly. Early detection of depression and cognitive impairment is important to delay disease progression. Therefore, we aimed to identify whether TL is associated with early subjective depressive symptoms and cognitive complaints among healthy elderly subjects. This study was a multicenter, outcome assessor-blinded, 24-week, randomized controlled trial (RCT). Measurement of questionnaire and physical activity scores and blood sample analyses were performed at baseline and after six months of follow-up in all study participants. Linear regression analyses were performed to identify whether early subjective depressive symptoms, cognitive complaints, and several blood biomarkers are associated with TL. Altogether, 137 relatively healthy elderly individuals (60–79 years old) were enrolled in this prospective RCT. We observed an approximate decrease of 0.06 and 0.11−0.14 kbps of TL per one point increase in the geriatric depression scale and cognitive complaint interview scores, respectively, at baseline and after six months of follow-up. We also found an approximate decrease of 0.08−0.09 kbps of TL per one point increase in interleukin (IL)-6 levels at baseline and after six months of follow-up. Our study showed that both early subjective depressive symptoms and cognitive complaints were associated with a relatively shorter TL in relatively healthy elderly individuals. In addition, based on our findings, we believe that IL-6 plays an important role in the relationship between shortening TL and early subjective depressive symptoms and cognitive complaints.

## INTRODUCTION

Telomeres are regions of tandem repeats of the base pairs TTAGGG located at chromosomal ends and undergo attrition with each division of somatic cells [1]. Therefore, telomere length (TL) is known as an indicator of replicative history and the replicative potential of cells. Attrition in TL has been reported be an important determinant of human aging not only at the cellular level but also systemically [2]. Older adults have increased risks of both depression and neurocognitive impairment [3]. Therefore, many studies have shown that TL is associated with depression or/and cognitive impairment in the elderly [4–9]. Individuals with late-life depression have a higher risk of developing cognitive impairment, significant functional disability, and medical comorbidity burden than that in young adults with depression [10]. This increase in aging-related somatic symptoms is purportedly a consequence of accelerated biological aging in individuals with depression [11]. Several hypotheses have been proposed to explain the emergence of a prematurely aged phenotype in late-life depression, such as glucocorticoid cascade dysregulation, increased allostatic load, and telomere shortening [10, 12].

We previously reported the randomized controlled trial (RCT) of the SoUth Korean study to PrEvent cognitive impaiRment and protect BRAIN health through lifestyle intervention in at-risk elderly people (SUPERBRAIN) [13, 14]. In that SUPERBRAIN studies, we assessed the feasibility of the facility-based multidomain intervention (FMI) and home-based multidomain intervention (HMI) programs in at-risk older Koreans. At the time of the study, questionnaire and physical activity scores and several blood biomarkers including TL were measured twice: at baseline and after six months of follow-up. As described above, TL is associated with geriatric diseases, and we wanted to evaluate possible associations between TL and geriatric diseases such as depression or/and cognitive impairment in the elderly using the data from our SUPERBRAIN study. Although there are many reports that TL shortening is related to major depression or substantial cognitive impairment and dementia in the elderly, there have been relatively few papers demonstrating that TL shortening is related to early depression symptoms or cognitive complaints in the healthy elderly. Early detection of depression and cognitive impairment is important and has the potential to significantly delay disease progression [15, 16]. Moreover, individuals with late-life depression exhibit increased concentrations of proinflammatory cytokines [17, 18]. Inflammatory cytokine IL-6 is also reported to be associated with depression [19–21].

Therefore, we aimed to identify whether TL is associated with not only major depression or substantial cognitive decline and dementia but also with early subjective depressive symptoms or/and cognitive complaints in relatively healthy elderly subjects. This was possible because the SUPERBRAIN study initially excluded elderly individuals with major depression, dementia, or severe cognitive impairment from the study. In addition, we analyzed the relationship between various biomarkers related to geriatric diseases and TL.

## RESULTS

### Characteristics of the study participants

Between May 29 and August 20, 2019, among 152 randomly assigned relatively healthy elderly participants (60–79 years old), 137 individuals who had TL measured both at baseline and after six months of follow-up were finally included in this randomized controlled prospective study. The average subject age was 70.8 years, and 74.5% of subjects were women (Table 1). The mean TLs at baseline and after six months of follow-up were 7.4 and 7.3 kbps, respectively. Based on a previous study, and considering our participants’ age range, the TL of our study participants were within the normal range [22]. More detailed information on the relationship between age and TL in the study participants is presented in Supplementary Figure 1. We observed that 24.8% of individuals had treatment histories for depressive symptoms. Further descriptive data are shown in Table 1.

**Table 1:** Characteristics of the study subjects.

**Characteristics**	**Total**
Number	137
Sex, female, n (%)	102 (74.5)
Age, mean ± SD, y	70.8 ± 4.8
Telomere length at baseline, mean ± SD, kbp	7.4 ± 1.1
Telomere length at baseline, median (IQR), kbp	7.4 (6.7–8.1)
Telomere length after 6 months of follow-up, mean ± SD, kbp	7.3 ± 1.1
Telomere length after 6 months of follow-up, median (IQR), kbp	7.3 (6.6–8.1)
BMI, mean ± SD, kg/m^2^	24.4 ± 2.8
Treatment history for depressive symptoms, n (%)	
Current	26 (19.0)
Past	8 (5.8)
No	103 (75.2)
Family history of dementia, n (%)	26 (19.0)
Hypertension, n (%)	68 (49.6)
Diabetes, n (%)	27 (19.7)
Hyperlipidemia, n (%)	71 (51.8)
Heart disease, n (%)	15 (10.9)
Stroke, n (%)	14 (10.2)

SD, standard deviation; IQR, interquartile range; BMI, body mass index.

### Association between TL and depressive episodes requiring treatment

**Figure 1:**
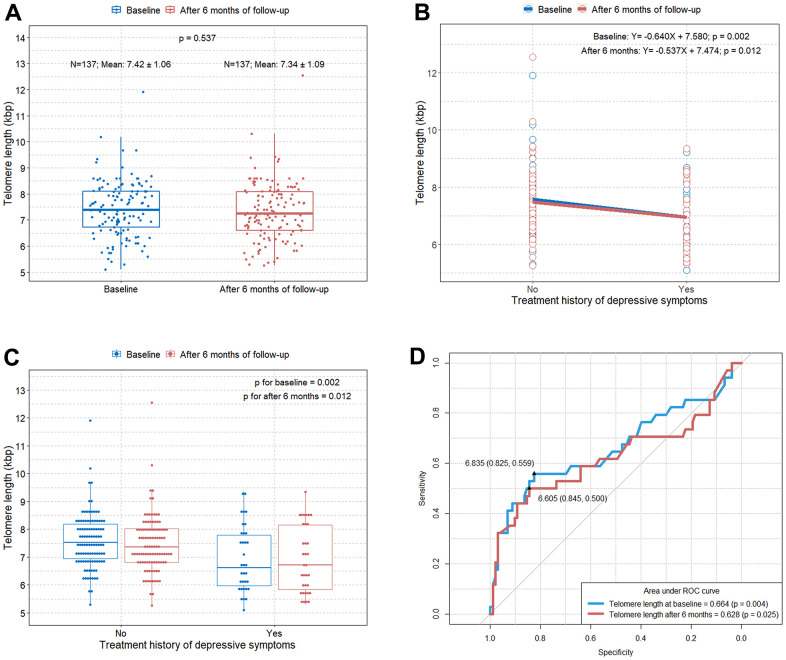
**Comparison of TL according to the treatment history for depressive symptoms at baseline and after six months of follow-up**. (**A**) Boxplot showing distributions of TLs in the study subjects at baseline and after six months of follow-up; (**B**) scatterplot with a linear regression line showing the difference in TL according to the treatment history for depressive symptoms at baseline and after six months of follow-up; (**C**) boxplot showing differences in TL according to the treatment history for depressive symptoms at baseline and after six months of follow-up; (**D**) ROC curve to identify the optimal cut-off values predicting depressive symptoms requiring treatment in elderly at baseline and after six months of follow-up. TL=telomere length; ROC=receiver operating characteristic.

The boxplot shows no significant difference in the TL between baseline and after six months of follow-up in the elderly subjects in this study (Figure 1A). Interestingly, we found that the TL was significantly shorter in individuals with a treatment history for depressive symptoms than in individuals without a treatment history for depression (Figure 1B, 1C). The optimal cut-off values of the TL for predicting treatment history of depressive symptoms at baseline and after six months of follow-up were 6.835 (area under the curve [AUC]=0.664; p=0.004) and 6.605 (AUC=0.628; p=0.025), respectively (Figure 1D).

### Correlation between TL and subjective depressive symptoms and cognitive decline

**Figure 2:**
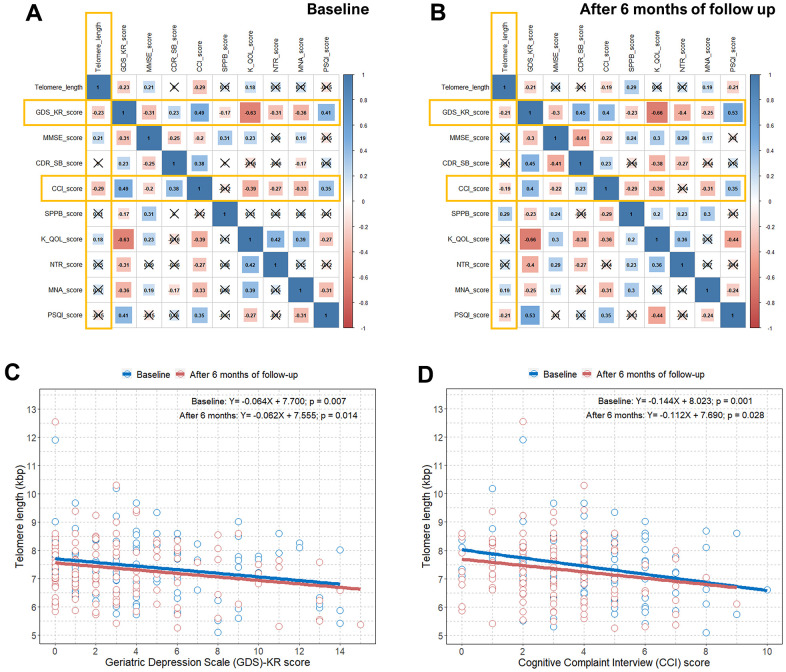
**Correlation plots between TL and questionnaire and physical activity scores and associations between TL and the GDS-KR and CCI scores**. (**A**) Pearson correlation coefficients and significance levels were calculated between TL and questionnaire and physical activity scores at baseline. The color-coordinated legend indicates the value and sign of Pearson’s correlation coefficient. The number in the box indicates Pearson’s correlation coefficient. Moreover, an x in the box indicates a p value≥0.005; (**B**) Pearson correlation coefficients and significance levels were calculated between TL and questionnaire and physical activity scores after six months of follow-up. The color-coordinated legend indicates the value and sign of Pearson’s correlation coefficient. The number in the box indicates Pearson’s correlation coefficient. Moreover, an x in the box indicates a p-value≥0.005; (**C**) scatterplot with a linear regression line showing the association between GDS-KR score and TL at baseline and after six months of follow-up; (**D**) scatterplot with a linear regression line showing the association between the CCI score and TL at baseline and after six months of follow-up. TL=telomere length; GDS-KR=Geriatric Depression Scale revised Korean version; CCI=Cognitive Complaint Interview.

We estimated the questionnaire and physical activity assessment scores at baseline and after six months of follow-up in all elderly subjects our study. There were statistically significant differences in the GDS-KR, CCI, SPPB, KQOL, and NTR scores between baseline and after six months of follow-up because 6-month intervention programs were implemented (Table 2). Unsurprisingly, there were strong inter-correlations between the questionnaire and physical activity assessment scores (Figure 2A, 2B). This is because cognitive decline (MMSE, CDR-SB, and CCI) and subjective depressive symptoms (GDS-KR) are interrelated, and they are known to be the major problems in the elderly [23]. Depression and cognitive impairment can subsequently affect physical activity (SPPB), quality of life (KQOL), nutrition status (NTR and MNA), and sleep quality (PSQI) in the elderly [23]. However, when we calculated the correlations between the questionnaire and physical activity assessment scores and TL, only the GDS-KR and CCI scores showed statistically significant associations with TL both at baseline and after six months of follow-up (an x in the box indicates a p-value≥0.005) (Figure 2A, 2B). The SUPERBRAIN study initially excluded elderly subjects with major depression, dementia, or severe cognitive impairment from the study; thus, the MMSE and CDR-SB, which are tools for dementia determination, were not associated with TL.

**Table 2:** Descriptive statistics of the questionnaire and physical activity scores and concentrations of several blood biomarkers.

	**Baseline**	**After 6 months of follow-up**	
Questionnaire and physical activity score, mean ± SD			p
GDS-KR	4.4 ± 3.8	3.5 ± 3.7	0.048
MMSE	27.8 ± 2.0	27.9 ± 2.0	0.627
CDR-SB	0.5 ± 0.5	0.5 ± 0.6	0.830
CCI	4.2 ±2.1	3.1 ± 1.8	<0.001
SPPB	11.3 ± 1.3	11.7 ± 0.8	0.002
KQOL	32.4 ± 6.0	34.3 ± 6.0	0.013
NTR	64.8 ± 9.8	69.3 ± 9.0	<0.001
MNA	12.0 ± 2.1	12.5 ± 2.0	0.071
PSQI	7.1 ± 3.8	6.9 ± 4.0	0.756
Concentration of blood biomarkers, mean ± SD, pg/ml			
BDNF	27612.5 ± 11784.2	28108.8 ± 13104.7	0.742
TREM2	4539.1 ± 2335.7	4150.3 ± 2312.7	0.167
IL-6	2.4 ± 2.9	2.1 ± 2.8	0.442
IL-18	160.8 ± 82.6	170.4 ± 87.5	0.350
IGF-1	87.5 ± 43.6	92.8 ± 51.2	0.360
YKL-40	78332.8 ± 74482.4	74351.6 ± 73748.3	0.657
TGF-β	36127.3 ± 9828.1	35042.9 ± 11172.5	0.394
TNF-α	0.6 ± 0.2	0.5 ± 0.2	0.045
VEGF	265.6 ± 211.2	269.3 ± 237.7	0.891
NfL	554.8 ± 330.1	468.8 ± 353.3	0.038
MCP-1	442.8 ± 314.0	415.4 ± 302.2	0.463

SD, standard deviation; GDS-KR, Geriatric Depression Scale revised Korean version; MMSE, Mini-Mental State Examination; CDR-SB, Clinical Dementia Rating-Sum of Boxes; CCI, Cognitive Complaint Interview; SPPB, Short Physical Performance Battery; KQOL, Korean Quality of Life; NTR, nutrition questionnaire for elderly; MNA, Mini Nutritional Assessment; PSQI, Pittsburgh Sleep Quality Index; BDNF, brain-derived neurotrophic factor; TREM2, triggering receptor expressed on myeloid cells 2; IL, interleukin; IGF-1, insulin-like growth factor 1; YKL-40, chitinase-3-like protein 1; TGF-β, transforming growth factor β; TNF-α, tumor necrosis factor α; VEGF, vascular endothelial growth factor; NfL, neurofilament light chain; MCP-1, monocyte chemoattractant protein 1.

We identified significant negative correlations between the GDS-KR and CCI scores and TL both at baseline and after six months of follow-up in Figure 2C, 2D. We observed an approximate decrease of 0.06 and 0.11−0.14 kbps in TL per one point increase in the GDS-KR and CCI scores, respectively, at baseline and after six months of follow-up. We also found a close relationship between subjective depressive symptoms and cognitive decline in relatively healthy elderly subjects, as shown in Supplementary Figure 2.

### Correlation between TL and IL-6

**Figure 3:**
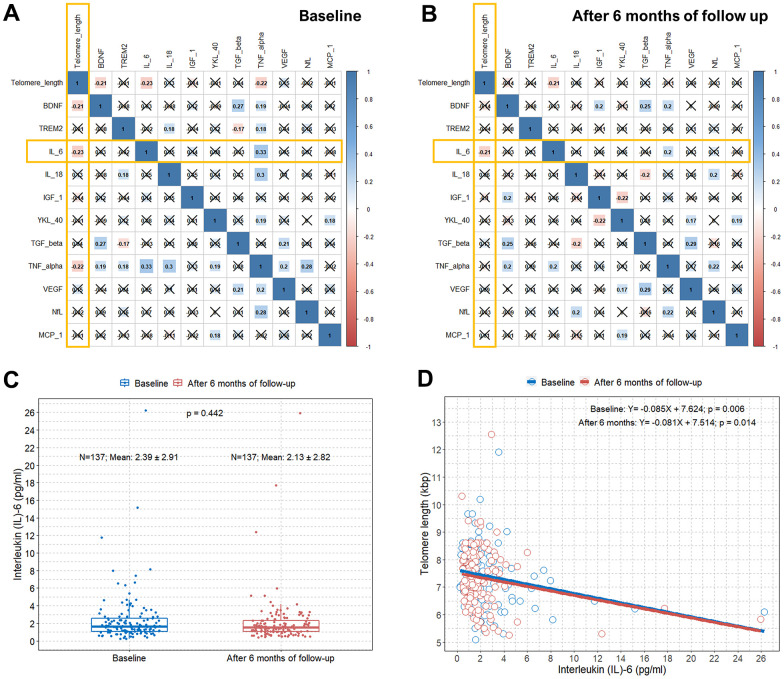
**Correlation plots between TL and concentrations of several blood biomarkers and associations between TL and serum IL-6 levels**. (**A**) Pearson correlation coefficients and significance levels were calculated between TL and concentrations of several blood biomarkers at baseline. The color-coordinated legend indicates the value and sign of Pearson’s correlation coefficient. The number in the box indicates Pearson’s correlation coefficient. Moreover, an x in the box indicates a p-value≥0.005; (**B**) Pearson correlation coefficients and significance levels were calculated between TL and concentrations of several blood biomarkers after six months of follow-up. The color-coordinated legend indicates the value and sign of Pearson’s correlation coefficient. The number in the box indicates Pearson’s correlation coefficient. Moreover, an x in the box indicates a p-value≥0.005; (**C**) Boxplot showing distributions of IL-6 levels in the study subjects at baseline and after six months of follow-up; (**D**) scatterplot with a linear regression line showing the association between IL-6 and TL at baseline and after six months of follow-up. TL=telomere length; IL-6= interleukin-6.

We also obtained values of several biomarkers at baseline and after six months of follow-up in all elderly subjects in the study. There were significant differences in the TNF-α and NfL values between baseline and after six months of follow-up (Table 2). Moreover, the mean IL-6 levels at baseline and after six months of follow-up were 2.4 and 2.1 pg/mL, respectively. Compared to a previous study, the IL-6 levels in our participants at similar ages were generally similar or slightly higher [24]. Visualized information on the relationship between age and IL-6 in our study participants is presented in Supplementary Figure 3. When we estimated the correlations between the values of these biomarkers and TL, only IL-6 was significantly correlated with TL both at baseline and after six months of follow-up (an x in the box indicates a p-value≥0.005) (Figure 3A, 3B). There was no significant difference in IL-6 values between baseline and after six months of follow-up (Figure 3C). We found an approximate decrease of 0.08−0.09 kbps of TL per one point increase in IL-6 at baseline and after six months of follow-up (B, −0.085; p=0.006; B, −0.081; p=0.014, respectively) (Figure 3D).

### Independent variables related to short TL in healthy elderly individuals

Multivariable linear regression analysis showed that higher GDS-KR and CCI scores and serum IL-6 levels were independently associated with a shorter TL both at baseline and after six months of follow-up in the relatively healthy elderly participants (Table 3).

**Table 3:** Multivariable linear regression of the telomere length according to the GDS-KR, CCI, or IL-6.

**Multivariable linear regression for telomere length**						
**Baseline**						
	GDS-KR*		CCI*		IL-6*	
**Variable**	**β (95% CI)**	**p**	**β (95% CI)**	**p**	**β (95% CI)**	**p**
Female	−0.073 (−0.518 to 0.372)	0.745	−0.070 (−0.507 to 0.366)	0.750	−0.217 (−0.663 to 0.229)	0.338
Age	0.005 (−0.036 to 0.045)	0.823	0.005 (−0.034 to 0.045)	0.792	0.004 (−0.036 to 0.043)	0.859
BMI	0.013 (−0.055 to 0.081)	0.702	0.019 (−0.048 to 0.086)	0.572	0.027 (−0.041 to 0.096)	0.431
Hypertension	−0.060 (−0.455 to 0.335)	0.764	−0.111 (−0.502 to 0.279)	0.574	0.045 (−0.344 to 0.435)	0.819
Diabetes	0.087 (−0.399 to 0.573)	0.723	0.081 (−0.393 to 0.556)	0.735	0.106 (−0.374 to 0.586)	0.663
Hyperlipidemia	−0.061 (−0.464 to 0.342)	0.764	−0.071 (−0.467 to 0.324)	0.721	−0.106 (−0.507 to 0.294)	0.600
Heart disease	0.059 (−0.586 to 0.703)	0.858	0.075 (−0.558 to 0.708)	0.815	0.057 (−0.584 to 0.697)	0.862
Stroke history	0.031 (−0.584 to 0.647)	0.920	0.019 (−0.585 to 0.624)	0.950	−0.040 (−0.653 to 0.574)	0.898
GDS-KR	**−0.062 (−0.112 to −0.011)**	**0.017**	N/A		N/A	
CCI	N/A		**−0.146 (−0.234 to −0.058)**	**0.001**	N/A	
IL-6	N/A		N/A		**−0.095 (−0.163 to −0.026)**	**0.007**
**After 6 months of follow-up**						
	**GDS-KR**		**CCI**		**IL-6**	
**Variable**	**β (95% CI)**	**p**	**β (95% CI)**	**p**	**β (95% CI)**	**p**
Female	0.065 (−0.389 to 0.520)	0.777	0.092 (−0.367 to 0.551)	0.692	0.087 (−0.369 to 0.542)	0.708
Age	0.004 (−0.037 to 0.045)	0.854	−0.004 (−0.045 to 0.037)	0.850	0.003 (−0.038 to 0.044)	0.889
BMI	0.015 (−0.055 to 0.084)	0.677	0.008 (−0.063 to 0.078)	0.830	0.027 (−0.044 to 0.098)	0.449
Hypertension	−0.249 (−0.663 to 0.165)	0.236	−0.197 (−0.608 to 0.213)	0.343	−0.131 (−0.533 to 0.270)	0.519
Diabetes	0.100 (−0.394 to 0.594)	0.690	0.105 (−0.394 to 0.603)	0.678	0.110 (−0.385 to 0.605)	0.661
Hyperlipidemia	0.053 (−0.361 to 0.466)	0.801	0.024 (−0.391 to 0.440)	0.907	0.000 (−0.413 to 0.414)	0.999
Heart disease	0.237 (−0.423 to 0.898)	0.478	0.302 (−0.367 to 0.970)	0.374	0.212 (−0.449 to 0.874)	0.527
Stroke history	−0.032 (−0.666 to 0.602)	0.921	−0.085 (−0.721 to 0.551)	0.466	−0.044 (−0.679 to 0.590)	0.890
GDS-KR	**−0.070 (−0.123 to −0.016)**	**0.011**	N/A		N/A	
CCI	N/A		**−0.120 (−0.228 to −0.012)**	**0.029**	N/A	
IL-6	N/A		N/A		**−0.086 (−0.155 to −0.017)**	**0.015**

GDS-KR, Geriatric Depression Scale revised Korean version; CCI, Cognitive Complaint Interview; IL-6, interleukin-6; CI, confidence interval; N/A, not available; p < 0.05 is shown in bold.

*Adjusted for sex, age, BMI, hypertension, diabetes, hyperlipidemia, heart disease, stroke history.

## DISCUSSION

We found that early and subjective depressive symptoms and cognitive complaints are associated with a relatively shorter TL among relatively healthy elderly individuals in the randomized controlled prospective SUPERBRAIN study. In addition, when analyzing the relationship between blood levels of several biomarkers and TL, a shorter TL was related to increased systemic levels of IL-6. It is possible that six months intervention programs affected the rate of TL shortening. However, according to our findings, there was no statistically significant difference in TL between baseline and after six months of follow-up (Figure 1A). We found that six months intervention programs affected the changes in GDS-KR and CCI scores and several biomarker values. Nevertheless, we believe that the results of this study showing that shorter TLs were still associated with increases in GDS-KR and CCI scores and systemic levels of IL-6 after six months of follow-up, as at baseline, are meaningful. To the best of our knowledge, this study is the first to show that TL is simultaneously associated with early and subjective depressive symptoms, cognitive complaints, and IL-6 in relatively healthy elderly individuals.

We measured scores related to subjective depressive symptoms using the 15-item GDS-KR and subjective cognitive decline with the CCI. The GDS is used widely as a screening instrument for early and subjective depressive symptoms in elderly [25, 26]. The CCI is a validated questionnaire for assessing subjective cognitive complaints and can be used in the early stages of dementia [27, 28].

Previous studies have reported that depression is associated with accelerated cellular aging and short TL [6–9]. It is well known that telomere shortening is a hallmark of biological age [2]. Major depressive disorder (MDD) is associated with accelerated cellular aging caused by the complex effects of several factors, such as the hypothalamic–pituitary–adrenal axis, brain-derived neurotrophic factor, mitochondrial DNA, telomerase, and inflammatory stress [7]. Among them, inflammatory pathways are known to be important factors influencing the connection between MDD and cellular aging [8]. A previous study described that IL-6 concentrations showed a significant inverse correlation with TL in the depressed group [6]. Our study also showed that a short TL was associated with subjective depressive symptoms and increased IL-6 levels among relatively healthy elderly individuals. Previous studies have also reported that IL-6 is associated with MDD [19–21]. Based on our findings, we believe that TL shortening in elderly is not only associated with advanced depression but also with early depressive symptoms. TL shortening is associated with increased IL-6 levels in the elderly, and we speculate that IL-6 may be a cytokine involved in depression from the early stages to advanced depression.

**Figure 4:**
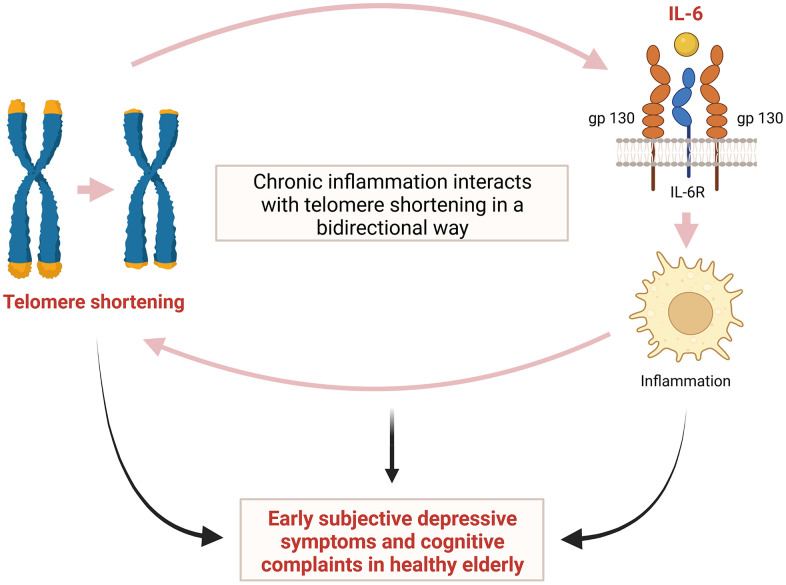
**Hypothetical interaction between telomere shortening and inflammation induced by IL-6 leading to early subjective depressive symptoms and cognitive complaints in relatively healthy elderly.** IL-6= interleukin-6.

Depression in the elderly is often associated with cognitive symptoms, which range from normal cognitive changes in aging to mild cognitive impairment to dementia [3, 23]. In our study, we also observed that a shorter TL was associated with both subjective depressive symptoms and early cognitive complaint in relatively healthy elderly individuals. It has been reported that shortening of TL in the elderly is associated with decreased cognitive function [4, 5]. The cumulative load of high IL-6 and high TNF-α can lead to an increased risk of short TL [29]. Therefore, as TL shortening is associated with decreased cognitive function in elderly, cognitive decline may be also associated with IL-6 in the elderly. A previous study reported an association between elevated IL-6 levels and risk of subsequent decline in cognitive function [30]. This study showed that IL-6 may be involved in early cognitive decline in elderly [30]. Taken together, we believe that our study shows the possibility that TL shortening may be associated with early depressive symptoms and cognitive complaints in the elderly. In addition, we hypothesized that IL-6 might serve as an intermediate link between TL shortening and early depressive symptoms and cognitive complaints in the elderly (Figure 4).

The precise mechanism underlying the correlation of shorter TL with depression, cognitive decline, and inflammatory cytokines remains unclear. However, although the complexity of the crosstalk between the inflammatory response and telomere shortening/dysfunction has not yet been fully revealed, evidence has led to the hypothesis that chronic inflammation interacts with TL in a bidirectional way [31]. Namely, in addition to the shortening effects of chronic low-grade inflammation on TL, cells with a shortened TL are more likely to secrete proinflammatory cytokines such as IL-6, thereby accelerating inflammation [31, 32]. This repeated process may cause cumulative structural and functional changes in the brain, leading to cognitive impairment and mood disorders including depression [31, 33]. It has also been suggested that the main mechanism of telomere shortening/dysfunction induced by chronic inflammation is oxidative stress [34]. Telomeres are known to be particularly sensitive to damage by oxidative stress and inflammation causing oxidative damage, and they are significantly less proficient in repair of DNA damage [35]. This telomere shortening and dysfunction induced by oxidative stress and inflammation may play an important role in both depression and cognitive impairment [31, 36]. A previous study showed that decreased expression of genes encoding the telomerase enzyme and important oxidative defense enzymes, such as superoxide dismutase (SOD1 and SOD2), catalase (CAT), and glutathione peroxidase (GPX1) can lead to shortening of TL [37]. It was also suggested that DNA methylation might play an important role in mediating the interaction among depression, inflammation, and TL [38]. The authors also identified a gene co-methylation module related to the connection between a history of depression and inflammatory conditions as well as between TL and serum IL-6 levels [38].

### Limitation of the study

First, this study was not originally designed to investigate the relationship between TL and subjective depressive symptoms and cognitive complaints in the elderly. Second, since this study was a feasibility RCT, it will be necessary to confirm the results of our study by performing large-scale RCT in the future. Third, the period between the two TL measurements was relatively short. Therefore, long-term changes in TL could not be analyzed. Fourth, the relatively small sample size in the present study may have influenced the results.

## CONCLUSIONS

In conclusion, we showed that both early subjective depressive symptoms and cognitive complaints in relatively healthy elderly individuals were associated with a relatively shorter TL in the randomized controlled prospective SUPERBRAIN study. In addition, a shorter TL was associated with increased IL-6 levels in our study participants. We believe that IL-6, an inflammatory cytokine, plays an important role in the relationship of shortening TL with early subjective depressive mood and cognitive complaints. Although the results will need to be verified through a large-scale RCT in the future, we believe that our findings will help prevent and treat depression and cognitive impairment in the healthy elderly.

## MATERIALS AND METHODS

### Study design and randomization

This study was a multicenter, outcome assessor-blinded, 24-week, RCT with a multi-arm parallel design performed in three hospitals and five public health centers across South Korea. The study participants were selected from among older adults who visited outpatient clinics or public health centers for memory complaints. As described in the Introduction, this study was originally designed to examine whether various outcomes were improved in the groups that received the FMI and HMI programs of the SUPERBRAIN compared with the control group. In a 1:1:1 ratio, participants were randomly assigned to the one of three groups: FMI, HMI, and control groups. Randomization was achieved through a permuted block randomization method, and the outcome assessors remained blinded to the assigned groups. Participants were instructed not to discuss their study involvement with the outcome assessor. We previously described the detailed study protocol [13, 14].

The study was performed in accordance with the International Conference on Harmonization Good Clinical Practices Guidelines. Institutional review boards approved the protocol and consent forms at each institution before the study began. Written informed consent was obtained from all potential study participants before their enrollment. This trial was registered with ClinicalTrials.gov (NCT03980392) [13, 14].

### Characteristics of the study participants and clinical assessments

As reported in our previous study [13, 14], the inclusion criteria of the study were as follows: (1) age between 60 and 79 years, (2) at least one modifiable dementia risk factor such as hypertension, diabetes mellitus, hyperlipidemia, obesity, abdominal obesity, metabolic syndrome, smoking, educational level ≤ 9 years, physical inactivity, and social isolation, (3) Mini-Mental State Examination (MMSE) Z score of ≥ -1.5, (4) ability to independently perform activities of daily living, as evaluated by Korean instrumental activities of daily living (K-IADL) [39] score < 0.4, (5) ability to read and write in Korean, as evaluated by a literacy test [40], and (6) accessibility to a reliable informant who could provide investigators with the requested information. The exclusion criteria were as follows: major psychiatric illness; substantial cognitive decline or dementia; other neurodegenerative diseases such as Parkinson’s disease; malignancy within the last five years; cardiac stent or revascularization within the previous year; serious or unstable symptomatic cardiovascular diseases; other serious or unstable medical disease such as acute or severe asthma, active gastric ulceration, severe liver disease, or severe renal disease; any conditions preventing cooperation as determined by the study physician; a significant laboratory abnormality that may result in cognitive impairment; inability to safely participate in the exercise program; or simultaneous participation in any other intervention trial [13, 14].

Treatment history of depressive symptoms was defined as currently receiving treatment or having received treatment in the past for depressive symptoms. Hypertension was defined as a prescription for antihypertensive medication(s) or a measured systolic blood pressure > 140 mmHg and diastolic blood pressure > 90 mmHg. Diabetes mellitus was defined as a prescription of insulin or oral hypoglycemic medications, a high plasma glucose level (≥ 126 mg/dL), or a high glycated hemoglobin level (≥ 6.5%) after eight hours of fasting. Hyperlipidemia was defined as a prescription for lipid-lowering medications or high levels of total cholesterol (≥ 200 mg/dL), low-density lipoprotein cholesterol (≥ 130 mg/dL), and triglycerides (≥ 150 mg/dL) and low levels of high-density lipoprotein cholesterol (< 40 mg/dL). Heart disease was defined as a history of an acute coronary event or arrhythmia including atrial fibrillation or use of heart-related drugs. Stroke was defined as a history of cerebral infarction or hemorrhage such as intracerebral hemorrhage and subarachnoid hemorrhage.

### TL assay and blood biomarkers

We obtained fasting blood samples from all participants at approximately 9 o’clock in the morning within four weeks before baseline and at the study endpoint. We previously described the detailed method of TL measurement as follows [41]: At baseline, whole blood from individuals was collected and separated into plasma and a buffy coat, and the leukocyte DNA was extracted using D-DEX™ IIb RBC lysis buffer and D-DEX™ IIb Cell lysis buffer (Intron, MA, USA). The DNA was then hydrated, and TLs were measured using a nonradioactive TeloTAGGG TL Assay (Roche Boehringer-Mannheim, Grenzach-Wyhlen, Germany). Briefly, 2–4 μg of DNA was fragmented and separated using agarose gel electrophoresis. The DNA fragments were then transferred to a nylon membrane (Millipore, Bedford, MA, USA) and incubated with digoxigenin (a digoxigenin-labeled probe), which specifically attaches to telomeric repeats. Next, the membranes were incubated with secondary antibodies conjugated with alkaline phosphatase. TLs were visually measured using chemiluminescence and an image analyzer (ImageQuant LAS 4000, GE Healthcare, Little Chalfont, UK). TLs were determined by comparing them to molecular weight standards.

Serum biomarkers such as brain derived neurotrophic factor (BDNF), triggering receptor expressed on myeloid cells 2 (TREM2), interleukin (IL)-6, IL-18, insulin-like growth factor 1 (IGF-1), YKL-40 (chitinase-3-like protein 1), transforming growth factor β (TGF-β), tumor necrosis factor α (TNF-α), vascular endothelial growth factor (VEGF), neurofilament light chain (NfL), and monocyte chemoattractant protein 1 (MCP-1) were also measured by a quantitative sandwich enzyme-linked immunosorbent assay [13].

### Questionnaire and physical activity scores

Questionnaire and physical activity scores were measured at baseline and after six months of follow-up in all elderly participants in the study. Subjective depressive mood was assessed by the 15-item Geriatric Depression Scale revised Korean version (GDS-KR) [42]. Cognition decline was determined by MMSE and Clinical Dementia Rating-Sum of Boxes (CDR-SB) [43, 44]. Subjective cognitive problems were assessed by cognitive complaint interview (CCI) [27]. We assessed participant physical functioning with the Short Physical Performance Battery (SPPB) and quality of life with the Korean version of Quality of Life-Alzheimer’s disease (KQOL) questionnaire [45]. Nutrition status was evaluated by a nutrition questionnaire for the elderly (NTR) [46] and Mini Nutritional Assessment (MNA) [47]. The Pittsburgh Sleep Quality Index (PSQI) was used to assess sleep quality.

### Statistical methods

Student’s t-test was performed to evaluate differences between values at baseline and after six months of follow-up. Box plots were used to visualize differences in TL and IL-6 levels at baseline and after six months of follow-up.

Receiver operating characteristic (ROC) curve analysis was used to determine the optimal cut-off TL that predicted depressive symptoms requiring treatment in the elderly. We used TL as the test variable and the treatment history of depressive symptoms as the state variable (dependent variable) in the ROC curve analysis. The optimal cut-off TL was defined as showing the shortest distance from the upper left corner (where sensitivity=1 and specificity=1).

Pearson’s correlation analysis was performed to evaluate the associations between TL and the questionnaire and physical activity scores and concentrations of blood biomarkers at baseline and after six months of follow-up.

Scatterplots with a regression line were constructed to determine whether TL was associated with GDS-KR and CCI scores and IL-6 levels. Multivariable linear regression analyses were also performed to identify whether GDS-KR and CCI scores and serum IL-6 levels were independently associated with TL. In addition to separately including GDS-KR, CCI score, and IL-6 level, covariates such as sex, age, BMI, hypertension, diabetes, hyperlipidemia, heart disease, and stroke history were entered into the multivariable model. For a comparison with the multivariable linear regression results, univariable linear regression analysis was also performed using the same variables as those used in the multivariable analysis (Supplementary Table 1).

A p-value < 0.05 was considered statistically significant. All statistical analyses were performed using R software version 4.1.2 and SPSS for Windows version 24.0 (IBM, Chicago, IL).

## Supplementary Material

Supplementary Figures

Supplementary Table 1
