# Supplementary material for: Relationship between telomere shortening and early subjective depressive symptoms and cognitive complaints in older adults
Source: Aging (Albany NY). 2023 Feb 17;15(4):914–31. doi: 10.18632/aging.204533 (PMC10008503; doi:10.18632/aging.204533)
Supplement: Supplementary Figures [file aging-15-204533-s001.pdf]

SUPPLEMENTARY FIGURES

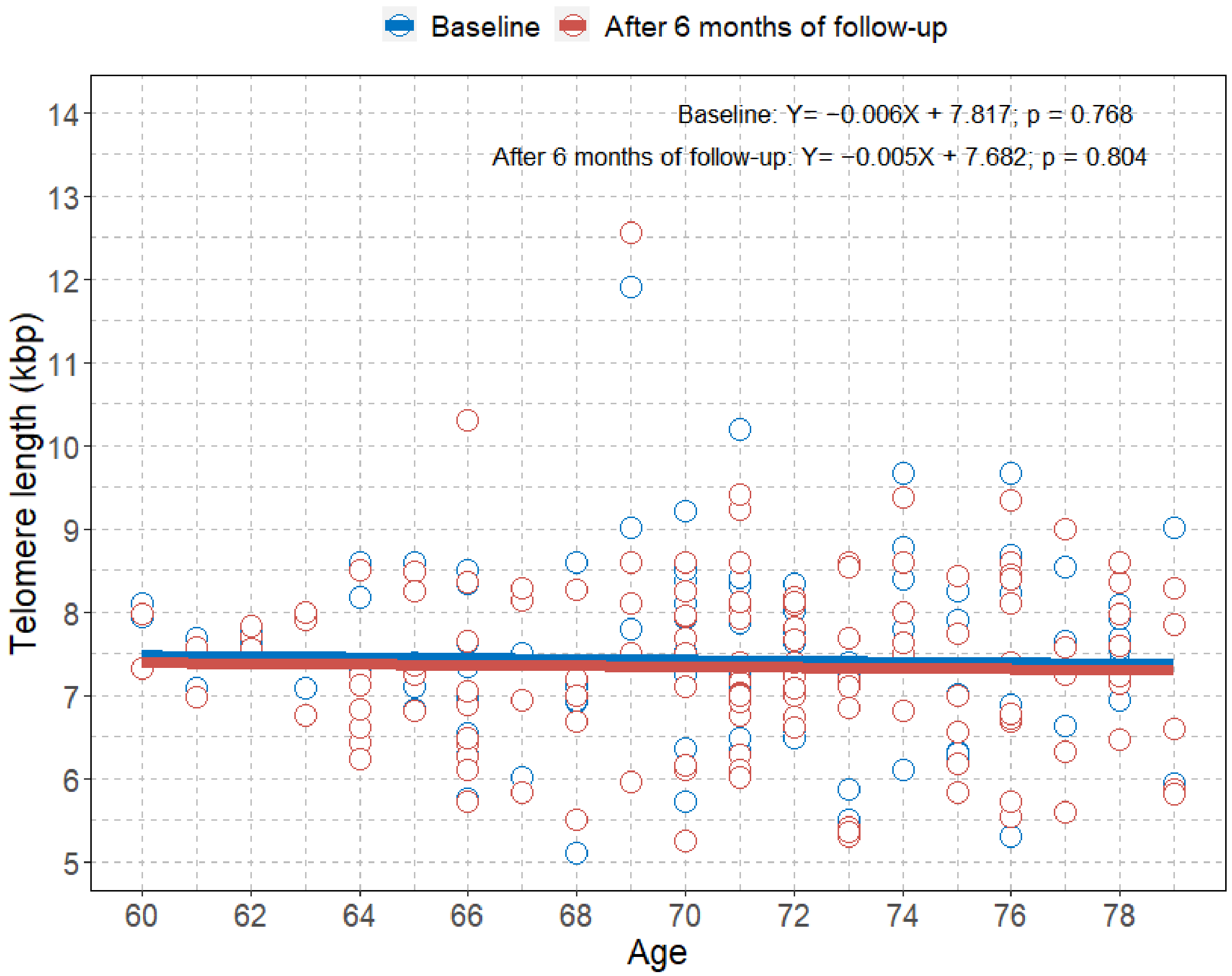

Supplementary Figure 1. Scatterplot with a linear regression line showing the association between age and the TL at baseline and after six months of follow-up. TL=telomere length.

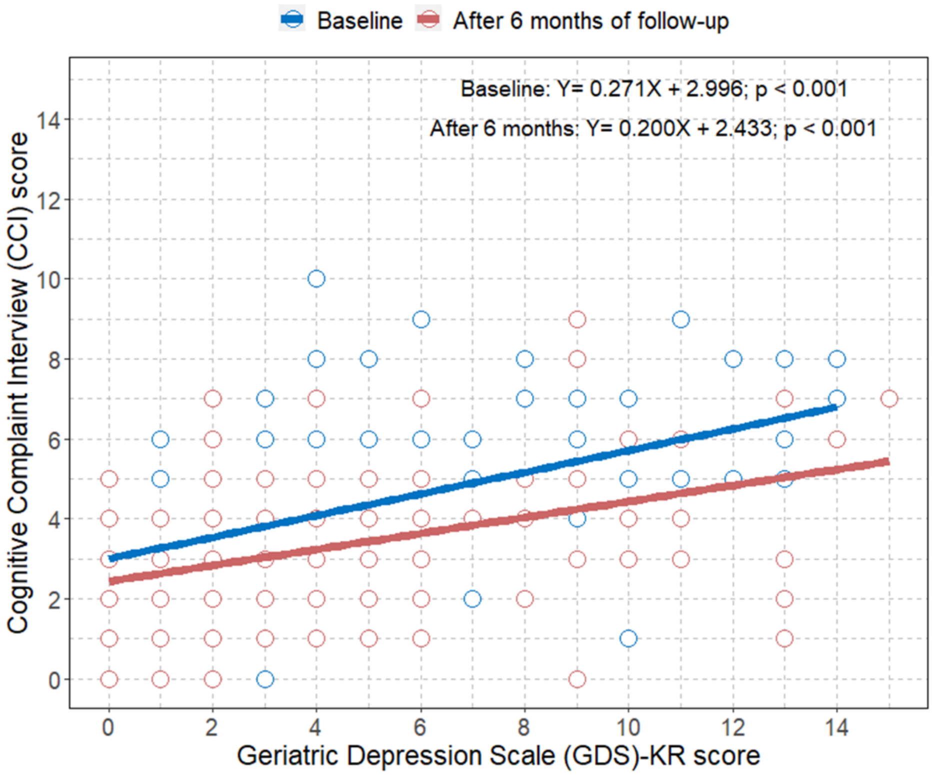

Supplementary Figure 2. Scatterplot with a linear regression line showing the association between the GDS-KR score and CCI at baseline and after six months of follow-up. GDS-KR=Geriatric Depression Scale revised Korean version; CCI=Cognitive Complaint Interview.

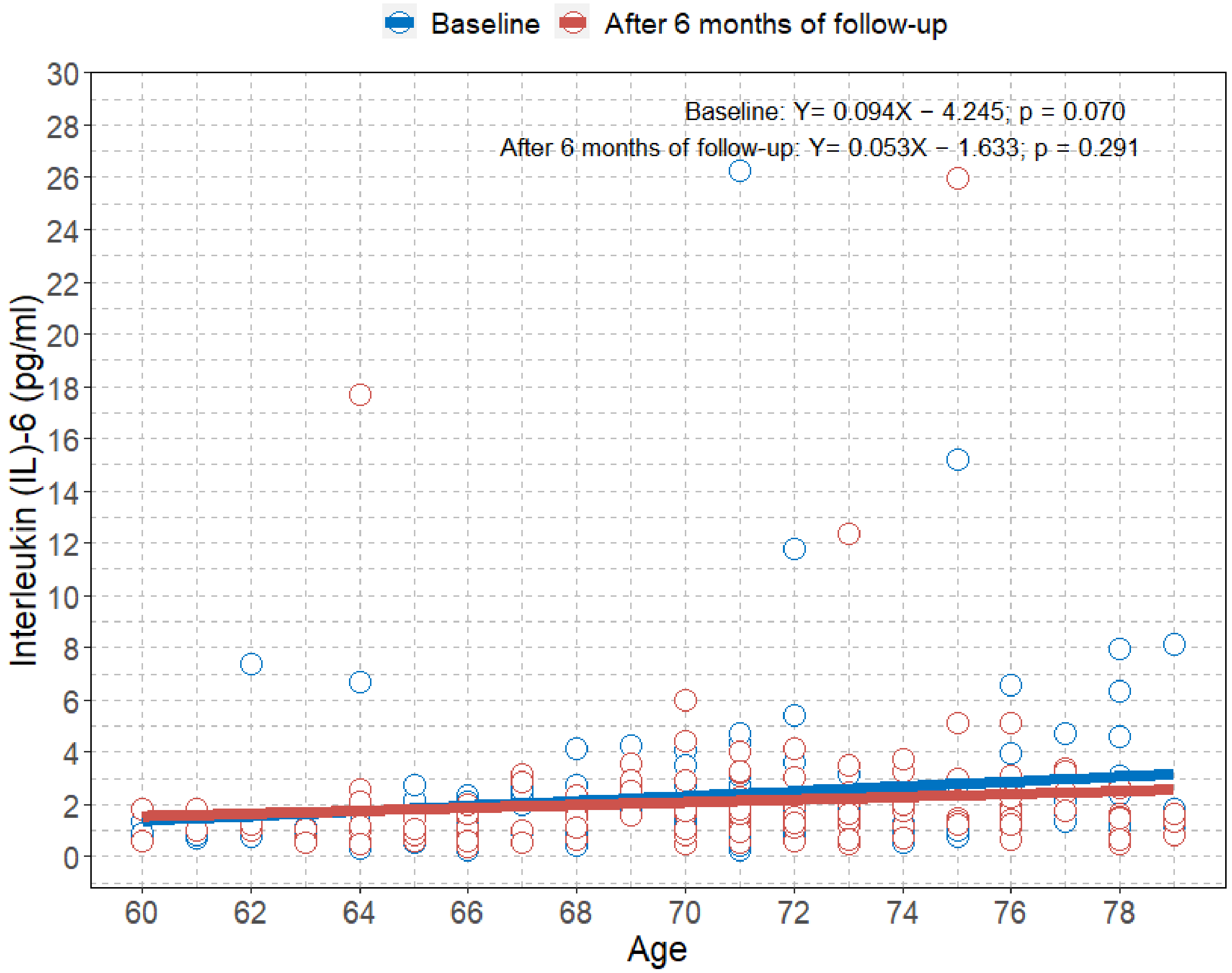

Supplementary Figure 3. Scatterplot with a linear regression line showing the association between age and IL-6 level at baseline and after six months of follow-up. IL-6= interleukin-6.
