# Supplementary material for: Relationship between telomere shortening and early subjective depressive symptoms and cognitive complaints in older adults
Source: Aging (Albany NY). 2023 Feb 17;15(4):914–31. doi: 10.18632/aging.204533 (PMC10008503; doi:10.18632/aging.204533)
Supplement: Supplementary Table 1 [file aging-15-204533-s002.pdf]

SUPPLEMENTARY TABLE

Supplementary Table 1. Univariable linear regression of the telomere length according to the same variables used in the multivariable analysis model.

Univariable linear regression for telomere length		
**Baseline**		
**Variable**	**β (95% CI)**	**p**
Female	−0.180 (−0.592 to 0.232)	0.390
Age	−0.006 (−0.043 to 0.032)	0.768
BMI	0.015 (−0.051 to 0.080)	0.656
Hypertension	0.027 (−0.336 to 0.390)	0.882
Diabetes	0.197 (−0.255 to 0.649)	0.390
Hyperlipidemia	−0.058 (−0.418 to 0.303)	0.753
Heart disease	0.123 (−0.454 to 0.699)	0.674
Stroke history	0.015 (−0.580 to 0.610)	0.960
GDS-KR	**−0.064 (−0.110 to −0.018)**	**0.007**
CCI	**−0.144 (−0.225 to −0.063)**	**0.001**
IL-6	**−0.085 (−0.145 to −0.024)**	**0.006**
**After 6 months of follow-up**		
**Variable**	**β (95% CI)**	**p**
Female	0.012 (−0.411 to 0.436)	0.954
Age	−0.005 (−0.043 to 0.034)	0.804
BMI	0.012 (−0.055 to 0.080)	0.721
Hypertension	−0.074 (−0.446 to 0.298)	0.695
Diabetes	0.186 (−0.278 to 0.649)	0.430
Hyperlipidemia	0.041 (−0.329 to 0.411)	0.828
Heart disease	0.205 (−0.386 to 0.796)	0.494
Stroke history	−0.118 (−0.728 to 0.492)	0.703
GDS-KR	**−0.062 (−0.111 to −0.013)**	**0.014**
CCI	**−0.112 (−0.212 to −0.012)**	**0.028**
IL-6	**−0.081 (−0.146 to −0.017)**	**0.014**

GDS-KR, Geriatric Depression Scale revised Korean version; CCI, Cognitive Complaint Interview; IL-6, interleukin-6; CI, confidence interval; $p < 0.05$ is shown in bold.
